# Enhancement attacks in biomedical machine learning

**Published:** 2023-01-05

**Authors:** Matthew Rosenblatt, Javid Dadashkarimi, Dustin Scheinost

**Affiliations:** 1 Department of Biomedical Engineering, Yale University; 2 Department of Computer Science, Yale University; 3 Department of Radiology and Biomedical Imaging, Yale School of Medicine

**Keywords:** machine learning, adversarial attacks, neuroimaging

## Abstract

The prevalence of machine learning in biomedical research is rapidly growing, yet the trustworthiness of such research is often overlooked. While some previous works have investigated the ability of adversarial attacks to degrade model performance in medical imaging, the ability to falsely improve performance via recently-developed “enhancement attacks” may be a greater threat to biomedical machine learning. In the spirit of developing attacks to better understand trustworthiness, we developed three techniques to drastically enhance prediction performance of classifiers with minimal changes to features, including the enhancement of 1) within-dataset predictions, 2) a particular method over another, and 3) cross-dataset generalization. Our within-dataset enhancement framework falsely improved classifiers’ accuracy from 50% to almost 100% while maintaining high feature similarities between original and enhanced data (Pearson’s *r*′*s* > 0.99). Similarly, the method-specific enhancement framework was effective in falsely improving the performance of one method over another. For example, a simple neural network outperformed LR by 50% on our enhanced dataset, although no performance differences were present in the original dataset. Crucially, the original and enhanced data were still similar (*r* = 0.95). Finally, we demonstrated that enhancement is not specific to within-dataset predictions but can also be adapted to enhance the generalization accuracy of one dataset to another by up to 38%. Overall, our results suggest that more robust data sharing and provenance tracking pipelines are necessary to maintain data integrity in biomedical machine learning research.

## 1 Introduction

Machine learning has demonstrated great real-world success across numerous fields. However, adversarial attacks, or data manipulations designed to alter the prediction [2], present a threat to real-world machine learning applications. Adversarial attacks include evasion attacks, where only test data are manipulated, or poisoning attacks, where the attacker may contribute manipulated test and/or training data [3]. Understanding adversarial attacks and developing corresponding defenses is crucial to the integrity of machine learning applications.

Machine learning is also becoming increasingly prevalent in biomedical research, including biomedical imaging. Previous studies of adversarial attacks in medical imaging have focused on clinical applications where a malicious party would be interested in altering the prediction outcomes for financial or other purposes. Most of these studies implemented evasion attacks [11,10], while a smaller subset used poisoning attacks [19,9]. An equally relevant yet understudied motivation in scientific machine learning is the feasibility of manipulating data to improve model performance falsely. For example, a malicious party might manipulate their data to improve model performance and thus make a paper more publishable or increase the valuation of a start-up. These data manipulations could waste grant money, misdirect future research directions of a given field, and potentially cause harmful public effects. One recent work showed that the performance of regression models using neuroimaging data could be falsely enhanced by injecting subtle associations into the data, labeled as “enhancement attacks” [23]. However, this enhancement framework is unsuitable for classification problems with discrete classes. Given the prevalence of classification problems in biomedical machine learning, understanding the potential to improve classification results through enhancement attacks is needed.

In this work, we first extend the enhancement attack framework to classification models (GOAL #1). Then, we present two other ways in which data can be enhanced with only subtle manipulations: falsely demonstrating that a particular method (e.g., type of machine learning model) outperforms another (GOAL #2) and falsely improving cross-dataset predictions (GOAL #3). Finally, we discuss the implications of enhancement in biomedical machine learning.

## 2 Methods

The enhancement attacks described in the remainder of this paper are heavily motivated by poisoning attacks, but they are distinct in both intention and attack capabilities. In poisoning attacks, particularly indiscriminate poisoning attacks, the attacker’s goal is to decrease the accuracy of all test samples by adding a small number of crafted training examples [6]. Despite the critical implications of poisoning attacks to real-world applications of machine learning, their importance in research-based machine learning is limited. A researcher would never want to manipulate data to make their model perform poorly. Enhancement attacks are based on a much more likely motivation behind manipulating data in research, which is to improve the performance of a model falsely.

Along with differences in motivation, the capabilities of the attackers in enhancement attacks are much greater than in poisoning attacks. In most studies of poisoning attacks, attackers are assumed to be capable of *adding* a small subset of points to the training data. In contrast, enhancement attackers can *modify* existing points. Moreover, poisoning attackers may have complete (“white-box”) or limited (“black-box” or “gray-box”) knowledge of the dataset and/or model [3]. In the research setting of enhancement attacks, the attacker has even greater knowledge than the traditional “white-box” setting, as they can modify the entire dataset, which may include both training and test data in the case of most within-dataset predictions. They can then publicly release this dataset such that the highly-performing model is computationally reproduced by others.

In the following sections, we considered three separate attacker goals: falsely enhancing 1) within-dataset classifier performance, 2) performance of one method over another, and 3) generalization of a model to an external dataset.

### GOAL #1: Within-dataset enhancement

Falsely enhancing within-dataset performance is the primary situation in which enhancement may occur. The motivation for studying the feasibility of within-dataset enhancement of machine learning comes from data manipulations in non-machine learning biomedical research. For instance, about 2% of papers investigated by [4] contained evidence of deliberate manipulations in biological images (e.g., western blots). However, possibilities of manipulations have not been studied for machine learning. In a hypothetical scenario, a biomedical researcher may enhance their dataset to improve prediction performance, thus leading to results that seem more impressive and interesting. The same researcher may then share this enhanced dataset, and others would computationally reproduce similar results, without any knowledge of the data manipulations that occurred.

The key idea behind within-dataset enhancement is to “push” the samples in the direction of a learned model to make the decision boundaries clearer and more consistent across all samples, thus improving performance. For a single held-out point, one may optimally change the classification by perturbing the point in the direction of ∇_*x*_*A*, where *A* can be a decision function or loss function. For example, in the case of linear support vector machine (SVM) or logistic regression (LR):

(1) $$X_{\text{held}-out,y=-1}\leftarrow X_{\text{held}-out,y=-1}-\epsilon *w$$

(2) $$X_{\text{held}-out,y=1}\leftarrow X_{\text{held}-out,y=1}+\epsilon *w$$

where *w* is a vector of model coefficients and *ϵ* is a scaling factor. Equations 1–2 would move the corresponding held-out points toward the correct side of the decision boundary. As summarized in Algorithm 1, first a model *f* is trained by holding one or numerous points out with K-fold partitioning. Then, the held-out point(s) are updated with ∇_*x*_*A* such that the model will predict them correctly, and this process repeats until all points are held out. Since learned model coefficients should be similar when only holding out a small fraction of the points, this method should push all points of a given class in a consistent direction. Eventually, when the enhanced dataset is released, an independent researcher would not notice any perturbations in the dataset but would falsely find higher performance.

### GOAL #2: Enhancement of a particular method

The motivation behind method enhancement would be to release a dataset and corresponding paper for which a neural network, for example, outperforms a simpler linear method. A second motivation could be for a company to demonstrate how their method (falsely) outperforms other methods to increase the valuation of a startup. Since a significant portion of biomedical machine learning research focuses on methods development, understanding the extent to which performance of one method can be enhanced over another is crucial.


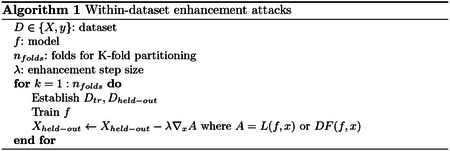


A roadblock to method-specific enhancement is that the gradients used in Equations 1–2 generally transfer well across model types [7], which would make this process ineffective in enhancing performance of a specific method over another. Transferability of attacks from a base classifier *f*_1_ to another classifier *f*_2_ is defined by [7] as how well an attack designed for *f*_1_ works on *f*_2_. In this case, we do *not* wish for the attacks to transfer between models. We want to find a new direction $g_{1}^{\prime }$ that enhances performance of *f*_1_ but does not affect *f*_2_. We achieve this by taking the component of *g*_1_ that is orthogonal to *g*_2_:

(3) $$g_{1}^{\prime }={\text{proj}}_{g_{2}^{⊥}}\left(g_{1}\right)$$

Furthermore, Equation 3 may not be sufficient to limit the performance of *f*_2_, since *f*_2_ can learn a new decision boundary after retraining. As such, we propose to include a term $g_{2}^{\prime }$ to suppress performance of *f*_2_:

(4) $$g_{2}^{\prime }={\text{proj}}_{g_{1}^{⊥}}\left(g_{2}\right)$$

Then, for a held-out sample, we can update it as follows to attempt to improve performance of *f*_1_ but not *f*_2_:

(5) $$x^{\prime }=x-\lambda \left(g_{1}^{\prime }-\eta g_{2}^{\prime }\right)$$

where *λ* and the suppression coefficient *η* control the influence of $g_{1}^{\prime }$ and $g_{2}^{\prime }$.

Similar to the model-based data enhancement, we split the data into *k* folds. For each partitioning, we train two models: 1) A model that we want to enhance (i.e., *f*_1_), and 2) a second model that we do not want to enhance (i.e., *f*_2_). Subsequently, Equations 3–5 are applied to update the held-out data, and the process is repeated until each sample is held out once.


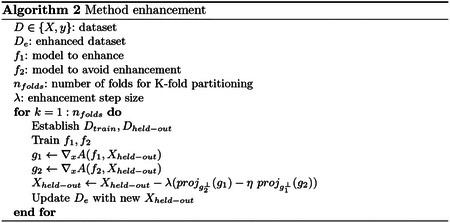


### GOAL #3: Cross-dataset enhancement

As previous sections on enhancement attacks were designed only to work for within-dataset predictions, requiring generalization of models to external datasets before deeming them “trustworthy” could be a potential defense against enhancement. Not only does generalization act as a defense against within-dataset enhancements, but it is also widely seen as the “gold-standard” for predictive models in science, as previous studies have highlighted that many within-dataset findings fail to generalize [17]. Consequently, we explored whether a dataset could be enhanced to falsely improve generalization performance.

In a hypothetical scenario, a researcher could plan to release a paper and dataset demonstrating prediction of a particular phenotype or outcome from imaging data. To make their results more impactful, the malicious researcher could download an external dataset (“generalization dataset”) and make minor changes to their dataset to falsely improve accuracy in the generalization dataset. Notably, no changes would be made to the generalization dataset. After publishing these seemingly favorable results and releasing the dataset, other researchers would computationally reproduce the good generalization performance.

Following previous works that used bilevel optimization in poisoning problems [6,18,7], we here use bilevel optimization for cross-dataset enhancement:

(6) $$\underset{\delta }{\text{min }}L\left(D_{g},f{\left(w\right)}^{*}\right)$$

(7) $$s.t.\;w^{*}\in \underset{w}{\text{arg min }}ℒ\left(D_{e},f(w)\right)$$

where *L* is the generalization loss, *ℒ* is the training loss, *D*_*g*_ is the generalization dataset, *D*_*e*_ is the enhanced training dataset, *f* is the model with parameters *w*, and *δ* is the perturbation applied to enhance the data. Notably, unlike previous poisoning problems, we seek to minimize the loss in the outer optimization problem (Equation 6). Since the training set is being altered, the loss function depends on the training point of interest. We can apply chain rule to compute the gradient of interest ∇_*x*_*A* [7]:

(8) $$\nabla_{x}A=\nabla_{x}L+\frac{\partial w^{T}}{\partial x}\nabla_{w}L$$

To solve this problem, one can replace the inner optimization of Equation 7 with the stationarity Karush-Kuhn-Tucker conditions and ultimately solve it as [7]:

(9) $$\nabla_{x}A=\nabla_{x}L-\left(\nabla_{x}\nabla_{w}ℒ\right){\left(\nabla_{w}^{2}ℒ\right)}^{-1}\nabla_{w}L$$

Instead of adding these gradients to poison, we will instead subtract them to enhance. For SVM, the formulation of the gradient in [2] is repeated below:

(10) $$\nabla_{x_{e}}A=\overset{m}{\underset{k=1}{\sum }}\left(M_{k}\frac{\partial Q_{se}}{\partial u}+\frac{\partial Q_{ke}}{\partial u}\right)\alpha_{e}$$

(11) $$M_{k}=-\frac{1}{\zeta }\left(Q_{ks}\left(\zeta Q_{ss}^{-1}-vv^{T}\right)+y_{k}v^{T}\right)$$

where *Q* is the label-annotated kernel matrix (i.e., *Q* = *yy*^*T*^ ◦ *K*), *s* indexes support vectors, *e* indexes the enhancement point, *k* includes all generalization points, *v* = *Q*^−1^*y*, $\zeta =y_{s}^{T}Q_{ss}^{-1}y_{s}$, *u* is the iteratively-updated enhancement direction, and *α*_*e*_ is the dual coefficient for the enhancement point.

For LR, the gradient described by [7] is repeated below for convenience:

(12) $$\nabla_{x_{e}}A=-{\left[\begin{array}{c} \nabla_{x_{e}}\nabla_{w}ℒ\\ Cz_{e}w \end{array}\right]}^{T}{\left[\begin{array}{cc} \nabla_{w}^{2}ℒ & XzC\\ CzX & C\sum_{i}^{n}z_{i} \end{array}\right]}^{-1}\left[\begin{array}{c} X(y\circ \sigma -y)\\ y^{T}(\sigma -1) \end{array}\right]C$$

where *C* is the regularization coefficient and *z* = *σ*(1 − *σ*) is the derivative of the logistic decision function.

For FFN, the bilevel optimization problem cannot be easily solved the same way as SVM and LR due to the complexity of the FFN. However, following the procedure of [18], we use back-gradient optimization [8,16] to more easily calculate the gradients for bilevel optimization. Essentially, back-gradient descent replaces the inner optimization problem (Equation 7) and is used to compute the gradient direction of the enhancement point $\nabla_{x_{e}}A$. In Algorithm 3, $\nabla_{x_{e}}\nabla_{w}ℒ$ and ∇_*w*_∇_*w*_*ℒ*(*x*′_*e*_, *w*_*t*_) are estimated with Hessian-vector products [18,20]. The sign of the resulting gradient in Algorithm 3 is then reversed for enhancement.


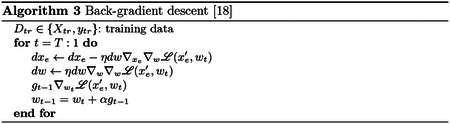



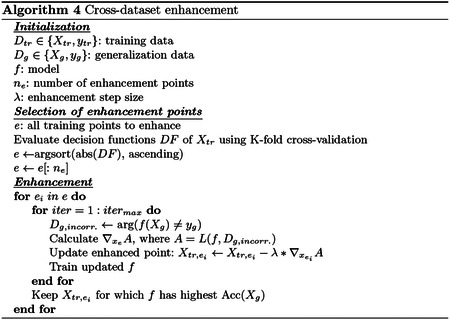


Unlike previous poisoning attacks, which only add new samples to the dataset, this cross-dataset enhancement attack alters existing samples. Our full implementation of cross-dataset enhancement is described in Algorithm 4. In brief, we altered training samples one-by-one, choosing the points on which the model was most “unsure” to alter first [15]. After training the initial model *f* on the unaltered data, we iteratively perturbed a point to minimize the loss on the generalization dataset. Notably, we only accounted for points which the model failed or had low confidence (i.e. close to decision boundary) when minimizing generalization loss. Unlike poisoning attacks where both a surrogate validation dataset and a test dataset are used to optimize model performance, the attacker has access to the full generalization dataset and thus a surrogate dataset is not necessary. After every enhancement point, the iteration with the highest accuracy is saved as the new “enhanced dataset” *D*_*e*_.

## 3 Experiments

### Datasets

Resting-state functional MRI data were obtained from the Adolescent Brain Cognitive Development (ABCD) [5], Human Connectome Project (HCP) [24], and UCLA Consortium for Neuropsychiatric Phenomics (CNP) [22] datasets. For all data, we performed motion correction, registration to common space, regression of covariates of no interest, temporal smoothing, and gray matter masking. Participants were excluded for excessive motion, missing behavioral data, missing task data (HCP only), or lack of full-brain coverage. Ultimately, 3251 participants remained in ABCD, 506 in HCP, and 245 in CNP. CNP was used for experiments in within-dataset enhancement and enhancement of a particular method, while HCP and ABCD were used in cross-dataset enhancement. For CNP, we classified participants based on their diagnoses, including no diagnosis (n=117), schizophrenia (n=46), bipolar disorder (n=44), and ADHD (n=38). For ABCD and HCP, we classified participants by self-reported sex, which was selected due to its availability across datasets and relative ease of prediction in fMRI data. All plots below were made with seaborn [13,25].

### GOAL #1: Within-dataset enhancement

We enhanced the CNP dataset for a classification problem with the following four classes: participants with 1) no diagnosis, 2) bipolar disorder, 3) schizophrenia, and 4) ADHD. We used linear SVM, LR [21], and a FFN as models. Our FFN consisted of three fully connected layers with the ReLU activation function. It was trained with the cross entropy loss and the Adam [14] optimizer, with a learning rate of 0.001 and batch size of 10 for 10 epochs.

**Fig. 1.**
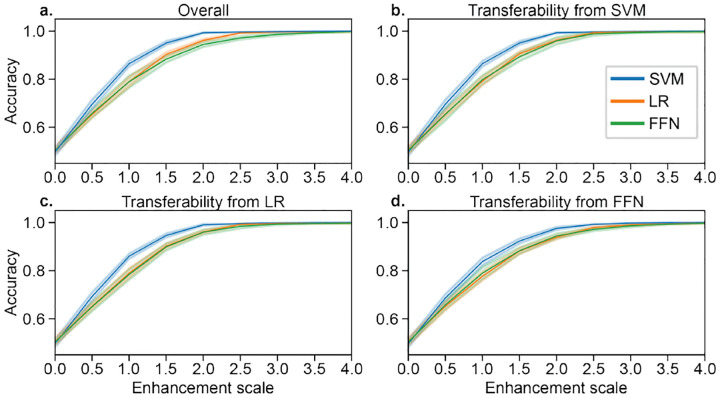
a) Model-based enhancement of SVM, LR, and FFN models for various enhancement scales. The enhancement scale is multiplied by the unit norm direction of the perturbation for each sample, where an enhancement scale of 0 reflects the original dataset. b-d) Transferability of enhancement between the three models. All accuracies were evaluated with 10-fold cross-validation, with error bars showing standard deviation across 10 random seeds.

Gradients were computed as the model coefficients in SVM and LR (linear models), while Pytorch’s autograd feature was used for the FFN. All gradients (i.e., ∇_*x*_*A* in Equation 8) were normalized to have a Frobenius norm of 1 and then multiplied by the corresponding enhancement scale in Figure 1. Enhancement brought prediction performance from ~50% to ~100% in all three models (Fig 1a), even though the feature values of the original and enhanced datasets are similar (*r* = 0.99). In addition to being effective for a particular model, the enhancement attacks transferred between each of the three models (Fig 1b–d).

### GOAL #2: Enhancement of a particular method

Since the enhancement attacks above transferred between models, we next investigated how enhancement may be targeted to a specific model. Because there are countless numbers of possible machine learning models, we selected three models to perform a case study: linear SVM, LR, and a FFN. We demonstrated the hypothetical scenario in which one may wish to perturb a dataset such that a FFN outperforms simpler methods like linear SVM and LR.

**Fig. 2.**
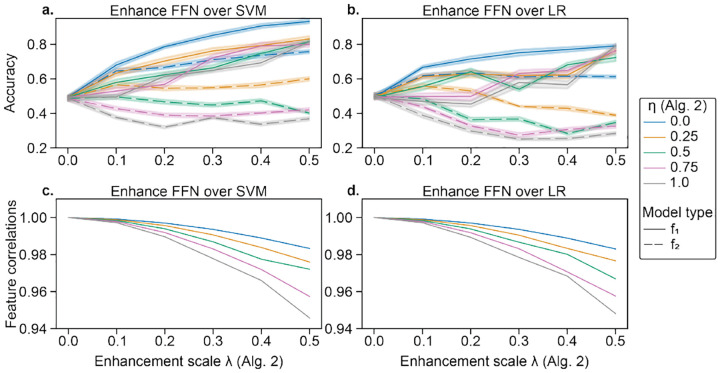
Enhancement of a FFN over a,c) SVM and b,d) LR in CNP. In a,b), data are enhanced with increasing *λ* (see Algorithm 2). Solid lines represent the accuracy for *f*_1_ (FFN), while dashed lines show the accuracy for *f*_2_ (SVM in a and LR in b). Error bars reflect standard deviation across 10 random seeds of K-fold cross-validation initialization seeds for FFN. Line color shows the suppression coefficient for *f*_2_, *η*. In c,d), the correlation between original and enhanced features is shown with increasing *λ*. The original and enhanced features are still highly correlated (*r*′*s* > 0.9).

For four-way classification in CNP, data were manipulated following Algorithm 2 to promote the performance of a particular method over another. We consider different enhancement scales *λ* for the classifier of interest (i.e., *f*_1_=FFN) and different suppression values *η* for the classifier which we do not wish to perform well (i.e., *f*_2_=SVM or LR). Despite no differences in the original dataset, FFN outperformed SVM and LR (Fig 2a–b), while maintaining high feature similarities (Fig 2c–d). The performance on *f*_2_ did generally increase, though less, as performance on *f*_1_ increased, but increasing the suppression coefficient *η* limited performance improvements of *f*_2_. Furthermore, attacks transferred between SVM and LR. For *f*_2_=SVM, *λ*=0.5, and *η*=1, accuracies for SVM and LR were 36.9% and 43.1% vs. 81.3% for FFN. For *f*_2_=LR, *λ*=0.5, and *η*=1, accuracies for SVM and LR were 25.8% and 28.4% vs. 79.3% for FFN.

### GOAL #3: Cross-dataset enhancement

**Fig. 3.**
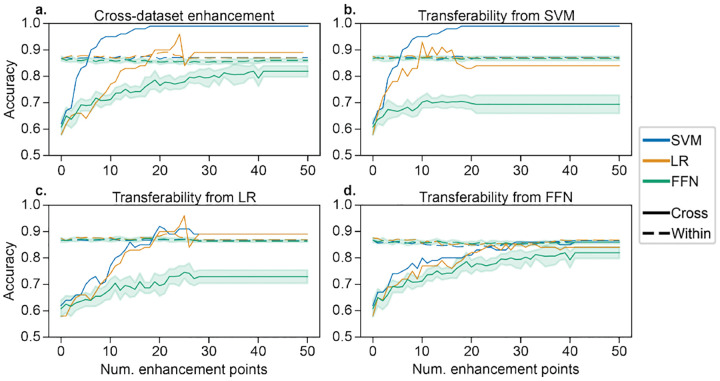
a) Cross-dataset enhancement of LR, SVM, and FFN models for prediction of self-reported sex, generalizing from HCP to ABCD. Up to 50 points were enhanced to improve cross-dataset prediction accuracy with *iter*_*max*_=20 and *λ*=0.15 (Alg 4). Solid lines show cross-dataset accuracy, and dashed lines show within-dataset accuracy. b-d) Transferability of enhancement in SVM, LR, and FFN models to the other models. Error bars show standard deviation across 10 random seeds to initialize the FFN.

For cross-dataset enhancement, we used binary classifiers of self-reported sex. Self-reported sex is suitable for cross-dataset evaluations because it is widely available in many datasets. HCP was the training dataset, and for our generalization dataset, we selected a random subset of 100 participants from ABCD. Feature selection was performed to select the top 10% most significantly different feature values in the training set. The FFN used sigmoidal activations and had one hidden layer with 100 neurons. It was trained with cross-entropy loss and a learning rate of 0.1. Both the model and the back-gradient descent procedure ran for 400 iterations. The 50 training points for which the model was least confident were perturbed sequentially. Average correlations between original and enhanced data were 0.991, 0.996, and 0.985 for SVM, LR, and FFN. Generalization accuracy increased by at least 18% after enhancement (Figure 3), and enhancement was most effective for SVM. Although accuracy should be monotonically increasing based on Algorithm 4, there was not a monotonic increase due to the re-selection of the 10% most significant features when evaluating the accuracy, which avoids leakage (*i.e*., different features may be selected in the original and enhanced data). In addition, enhancement of cross-dataset predictions did not affect within-dataset performance (dashed lines in Figure 3), making cross-dataset enhancement even more inconspicuous.

Furthermore, we investigated how cross-dataset enhancement attacks may transfer to other models. Enhancement points were optimized on SVM (Figure 3a), LR (Figure 3b), and FFN (Figure 3c) and then evaluated with the other two models. SVM and LR exhibited a moderate degree of transferability between each other, but neither had strong transferability to FFN. However, the FFN-optimized enhancement points did transfer to SVM and LR. Finally, using SVM models as an example, we tested the sensitivity of cross-dataset enhancement to the number of generalization samples. We randomly selected a subset of 100, 200, 400, or 800 generalization samples and repeated this ten times. The best cross-dataset accuracies (standard deviations across random seeds in parentheses) after enhancing up to 100 training points were 0.983 (0.015), 0.961 (0.020), 0.896 (0.023), and 0.815 (0.016) for 100, 200, 400, and 800 generalization points, respectively. These ranged from 17% to 31% better than baseline values, and enhancement effectiveness decreased with more generalization points.

## 4 Discussion

In this work, we first adapted enhancement attacks for classifiers, demonstrating that a four-way classification task went from near-chance performance to over 99% accuracy while the original and enhanced data remained highly similar (*r* > 0.99). We then enhanced the performance of a specific model over another. In the best case, a FFN outperformed LR by up to 50% in the enhanced dataset, despite no differences in original performance and high similarity between original and enhanced data (*r* = 0.95). Finally, while cross-dataset generalization was previously suggested as a possible way to mitigate data enhancement, we showed that generalization accuracies could be enhanced from ~60% to ~80–100% while changing at most 50 points in the training dataset.

Although our analysis was restricted to functional neuroimaging, these problems extend to the greater biomedical machine learning communities, where many view data and code sharing as the panacea for trustworthiness. In adversarial attacks, the attacker has only limited access to the model and data. However, given the unrestricted access of the attacker in enhancement attacks, the most reasonable defense is data provenance tracking, such as DataLad [12]. We recognize that most researchers would be ethically opposed to enhancing their data. Yet, plenty of fraud occurs in scientific journals and clinical trials [1,4], suggesting that enhancement attacks could become a problem in scientific machine learning. Due to the feasibility of enhancement attacks, better data provenance is necessary to ensure trustworthy biomedical machine learning.

### Limitations

We investigated enhancement only in functional neuroimaging. Future work should expand these concepts to other disciplines. These other disciplines may have important differences, such as sample sizes or data dimensionality. In addition, future work should evaluate within-dataset enhancement of more complex architectures. Finally, whether one complex architecture can be enhanced over another with subtle differences (i.e., using method enhancement) remains to be seen.
